# The impact of agricultural insurance on consumer food safety: empirical evidence from provincial-level data in China

**DOI:** 10.3389/fnut.2024.1392711

**Published:** 2024-05-15

**Authors:** Peiheng Ruan, Sihan Yin, Yongchang Zhang

**Affiliations:** ^1^School of Economics and Management, Wuhan University, Wuhan, China; ^2^Wuhan College, Wuhan, China

**Keywords:** agricultural subsidies, agricultural insurance, food safety, agricultural technological innovation, environmental pollution, consumer confidence

## Abstract

In the exploration of the efficacy of agricultural subsidy policies, agricultural insurance, as a key element of this policy system, has garnered widespread attention for its potential impact on consumer food safety. This paper delves into the influence of agricultural insurance on the safety of food consumed by individuals, based on provincial panel data in China from 2011 to 2021. The findings indicate that agricultural insurance significantly reduces the incidence of foodborne disease and enhances food safety. Mediating effect tests reveal that agricultural insurance effectively boosts food safety through two key pathways: promoting innovation in agricultural technology and reducing environmental pollution. Moreover, the analysis of moderating effects highlights that increased consumer confidence positively enhances the impact of agricultural insurance. Heterogeneity tests further show that in the provinces with higher levels of agricultural development and stronger government support for agriculture, the role of agricultural insurance in improving food safety is more pronounced. This research not only empirically verifies the effectiveness of agricultural insurance in enhancing food safety but also provides robust theoretical support and practical guidance for the precise formulation and effective implementation of agricultural subsidy policies, particularly agricultural insurance policies, offering significant reference value for policy-makers.

## 1 Introduction

As the process of globalization continues to accelerate, food safety has become a severe challenge in the global public health sector, eliciting widespread concern worldwide. The complexity of food safety issues not only involves every link in the chain of food production, processing, distribution, and consumption but is also closely related to consumer health and life safety (1, 2). According to the Food and Agriculture Organization (FAO) and the World Health Organization (WHO), approximately 600 million people suffer from foodborne diseases globally each year, resulting in approximately 420,000 deaths, highlighting the significant threat of food safety to human health. Furthermore, food safety is directly linked to the stable development of a country’s economy and the enhancement of social welfare, thus, it has become an important indicator to measure the comprehensive development level of a country or region (3). Among various factors, agriculture, as the starting point of the food supply chain, plays a decisive role in ensuring food safety through the stability and sustainability of its production processes. Agricultural subsidies, as a significant policy tool, support agricultural production directly or indirectly, with agricultural insurance policies being considered effective means to reduce agricultural production risks and ensure the stability and sustainability of agricultural production (4). Despite the widespread belief in the positive impact of agricultural insurance on enhancing food safety, there are still relatively few empirical research on how agricultural insurance actually affects food safety (5), especially in a country like China where agricultural production and consumption are extremely important. This research gap limits our comprehensive understanding and assessment of the effects of agricultural insurance policies and also constrains the potential of agricultural insurance in enhancing global and regional food safety.

In China, the implementation of agricultural insurance policies has become one of the key strategies for supporting agricultural development, promoting rural stability, and ensuring food safety (5). In recent years, with the Chinese government’s increasing focus on the agricultural assurance system, agricultural insurance, as a core tool for agricultural risk management, has seen significant enhancements in its coverage and protection capabilities (6). The government has vigorously promoted the widespread adoption and application of agricultural insurance through measures such as providing financial subsidies, optimizing insurance product design, and expanding insurance coverage types (7). Existing literature points out that agricultural insurance, by mitigating risks to agricultural production from unforeseen factors such as natural disasters, helps to ensure the continuity and stability of agricultural production, thereby indirectly affecting the safety of food production and supply (8, 9). However, while this potential mechanism is theoretically acknowledged, empirical research on how agricultural insurance impacts food safety through specific pathways remains scarce in the Chinese context, limiting our ability to fully understand and evaluate the effects of agricultural insurance policies. Therefore, a thorough exploration of the current situation of China’s agricultural insurance policies and their specific impact mechanisms on food safety not only has significant practical significance but also holds substantial policy value for optimizing agricultural insurance policy design, improving policy implementation efficiency, and enhancing the food safety assurance system.

In the field of agricultural insurance, existing literature mainly focuses on exploring how agricultural insurance affects the stability of farmers’ income (10–13), mitigates risks from natural disasters (14–17), and promotes agricultural economic development (18–20), including green economy development (21–23). These findings undoubtedly provide important reference values for the formulation and implementation of agricultural insurance policies. However, these studies often focus on the economic and environmental benefits of agricultural insurance, with relatively less discussion on how agricultural insurance impacts food safety. Especially in an agricultural powerhouse like China, despite the gradually recognized role of agricultural insurance in enhancing agricultural production stability and promoting environmental protection in agriculture, the link and mechanism of action between agricultural insurance and food safety still lack sufficient empirical research support. Particularly, discussions on how agricultural insurance impacts food safety through indirect pathways, such as promoting technological innovation and improving environmental quality, are rare in the literature. Although some studies mention the positive impact of agricultural insurance on various links of the food supply chain, they often lack sufficient empirical evidence to support their conclusions or fail to systematically analyze the direct and indirect connections between agricultural insurance and food safety. Additionally, existing literature overlooks how changes in consumer confidence in food safety affect the role of agricultural insurance in food safety and how regional differences in policy implementation may impact strategy effectiveness. These research gaps highlight an essential fact: while agricultural insurance is theoretically considered as an important policy tool to enhancing food safety, empirical research on its specific mechanisms of action, effect differences, and policy implementation in China needs further deepening and expansion.

Given the limitations of existing literature, this study utilizes annual panel data from 30 provinces in China between 2011 and 2021 to conduct an empirical analysis aimed at deeply exploring the impact of agricultural insurance on food safety. Initially, this research assesses the direct effects of agricultural insurance on enhancing consumer food safety, and then investigates the specific mechanisms through which agricultural insurance affects food safety via two key pathways: agricultural technological innovation and environmental pollution reduction. Furthermore, this paper explores how consumer confidence moderates the impact of agricultural insurance on food safety. Lastly, considering that the level of agricultural development and government support might influence the effectiveness of agricultural insurance, we further examine the heterogeneous effects of agricultural insurance on consumer food safety from these two perspectives. Through these detailed analyses, this study gains a more comprehensive and in-depth understanding of the effectiveness of agricultural insurance policies, offering more precise and effective advice for policymakers.

The main contributions of this paper are manifested in two aspects: On the theoretical level, through systematic empirical research, this paper fills the research gap in existing literature regarding how agricultural insurance impacts food safety. Specifically, it clearly reveals the specific mechanisms by which agricultural insurance enhances food safety through promoting agricultural technological innovation and reducing environmental pollution. Additionally, this study is the first to examine the moderating role of consumer confidence in the relationship between agricultural insurance and food safety, providing a new theoretical perspective and methodological support for understanding the complex impact effects of agricultural insurance policies across different socio-economic backgrounds. On the practical level, the findings of this study offer an empirical foundation for formulating and optimizing policies aimed at enhancing food safety through agricultural insurance. In particular, by identifying the key factors and pathways affecting the effectiveness of agricultural insurance, this study provides a basis for policymakers to design more targeted policy measures, thereby helping to improve the effectiveness and efficiency of policies and further promoting food safety and sustainable agricultural development.

## 2 Theoretical analysis and research hypotheses

Agricultural insurance, as an important form of risk management, plays a crucial role in agricultural production (24, 25). By providing risk protection to agricultural producers, agricultural insurance effectively reduces losses caused by natural disasters such as floods, droughts, and pestilences, offering financial compensation to agricultural producers when facing these adverse conditions (14). This economic compensation mechanism ensures that farmers can continue or quickly resume production activities after experiencing natural disasters, thereby reducing the possibility of production interruptions caused by disasters (26). Moreover, the presence of agricultural insurance also promotes farmers’ risk management of future uncertainties, enhancing their risk adaptation capacity. This not only guarantees the continuity and stability of agricultural production but also indirectly improves agricultural production efficiency and sustainability (27, 28). Stable and sustainable agricultural production is vital for maintaining the continuity of the food supply chain, helping to reduce the problems of food shortages caused by production fluctuations or interruptions (29), thus effectively avoiding the risk of foodborne diseases and enhancing the safety and reliability of food.

On this basis, the impact of agricultural insurance on agricultural production methods cannot be ignored. By increasing the stability of agricultural production, agricultural insurance motivates farmers to adopt safer, environmentally friendly agricultural production technologies and methods (30). These production methods not only help to improve the efficiency and quality of agricultural production but also reduce the potential for harmful substance residues in agricultural production, such as reducing the excessive use of chemical fertilizers and pesticides, promoting the development of organic and ecological agriculture (29, 31). This promotion of sustainable agricultural practices is not only beneficial for environmental protection but also directly enhances food safety, as these practices reduce the potential for harmful substances in food, thereby ensuring consumer health (32).

Furthermore, as an effective risk management tool, agricultural insurance, by ensuring the stability of agricultural production, indirectly promotes the safety management of the entire food supply chain (33). With the protection of agricultural insurance, agricultural producers are more likely to comply with the standards and regulations for food safety production, implement Good Agricultural Practices (GAP), and take necessary food safety control measures (34, 35). These measures can effectively manage and control safety risks in the food production process, ensuring that every link in the food chain from field to table meets safety standards, thereby directly enhancing food safety.

Based on the above analysis, we propose the Hypothesis 1: Agricultural insurance can reduce the incidence rate of foodborne diseases and enhance the safety of consumer food consumption.

Agricultural technological innovation plays a crucial role in enhancing agricultural production efficiency and food safety. In recent years, with the development of biotechnology, precision agriculture technology, and environmental-friendly agricultural practices, agricultural production modes is undergoing fundamental transformations (36, 37). The application of these new technologies not only improves the growth conditions of crops, increasing yield and quality, but also effectively reduces the use of chemical pesticide, thereby significantly lowering the potential for harmful residues in food and the risk to public health (38, 39). Against this backdrop, agricultural insurance, as a risk mitigation mechanism, plays an important role in promoting agricultural technological innovation. By providing financial compensation for economic losses, agricultural insurance reduces the economic risks farmers might face when trying new technologies (40). The existence of this economic safety net significantly increases farmers’ willingness to accept new technologies, stimulating their enthusiasm to adopt new technologies to improve production efficiency and food safety. Furthermore, agricultural insurance also promotes investment in agricultural technological innovation by research institutions and enterprises, accelerating the research, development, and promotion process of new technologies (41). This series of activities fosters the development of agricultural technological innovation, thereby enhancing the safety of the entire food supply chain.

Simultaneously, the environmental impact of agricultural production is a key factor affecting food safety. Inappropriate agricultural activities, such as the excessive use of chemical fertilizers and pesticides, can lead to soil degradation, water pollution, and a reduction in biodiversity, ultimately affecting every link in the food chain and posing a threat to consumer health (42, 43). In this regard, agricultural insurance encourages farmers to adopt environmentally friendly production methods, such as organic farming, water-saving irrigation techniques, and natural pest and disease management, mitigating the negative impact of agricultural production on the environment (44). Insurance companies offer more favorable insurance terms to farmers who adopt sustainable agricultural production practices, further incentivizing a shift toward environmentally friendly production methods (45). This shift helps to maintain the health of the ecological environment, reducing potential pollution and risks during the food production process, thereby ensuring food safety.

Thus, agricultural insurance, by promoting agricultural technological innovation, reduces the use of chemical pesticides, lowering the risk of harmful residues in food. Simultaneously, by encouraging the adoption of environmentally friendly agricultural production methods, agricultural insurance helps to reduce the negative impact of agricultural production on the environment, protecting natural resources and thereby enhancing the safety of the entire food chain.

Hence, we propose the Hypothesis 2: Agricultural insurance can effectively enhance food safety through two pathways by improving the innovation of agricultural science and technology and reducing environmental pollution.

In contemporary economic research, consumer confidence is regarded as an important indicator of market behavior, particularly in the realm of food safety, where consumer confidence in food directly affects their purchasing decisions and consumption behaviors (46, 47). Signaling theory provides a robust framework to explain how agricultural insurance can act as a positive signal, influencing consumer confidence in food safety. From this theoretical perspective, agricultural insurance is not only a risk management tool but also an information transmission mechanism, conveying to the market the agricultural producers’ commitment and assurance toward food safety (48). Consumer confidence in food originates from trust in the safety of food, which is based on understanding the food production and processing procedures and awareness of food quality control standards. As a risk mitigation mechanism, the mere presence of agricultural insurance transmits a message to consumers that agricultural producers have taken precautionary and protective measures against potential risks (49). This transmission of information helps to reduce consumers’ concerns about unknown risks to food safety, increasing their confidence in the quality and safety of agricultural products.

Furthermore, when consumer confidence in food is bolstered, they are more likely to support and choose agricultural products that have taken additional safety measures, such as participating in agricultural insurance programs (50, 51). This change in consumer behavior, in turn, encourages more agricultural producers to participate in agricultural insurance programs, as they see the economic incentives of meeting consumer demand by enhancing food safety (52). Therefore, the enhancement of consumer confidence not only directly raises expectations for food safety but also promotes the role of agricultural insurance in improving food safety through market mechanisms.

In summary, the enhancement of consumer confidence has a significant positive effect on the impact of agricultural insurance on food safety. This effect is manifested both in directly boosting consumer confidence in food and in encouraging more agricultural producers to adopt agricultural insurance through market feedback mechanisms, thereby indirectly raising the overall level of food safety.

Based on this, we propose the Hypothesis 3: The enhancement of consumer confidence has a positive promotional effect on the impact of agricultural insurance.

**Figure 1:**
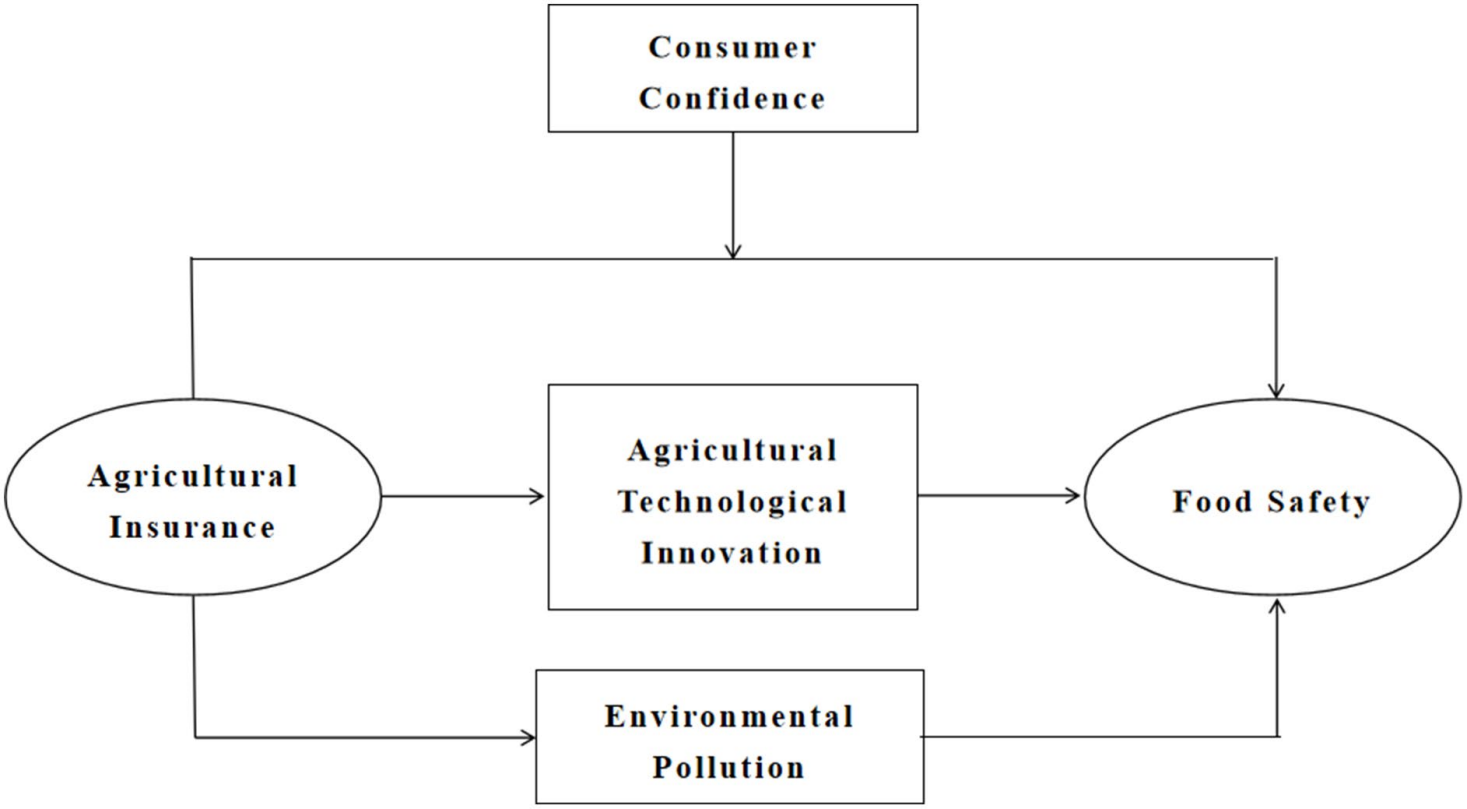
Theoretical model diagram.

The theoretical model diagram of the above research hypotheses is shown in Figure 1.

## 3 Research design

### 3.1 Empirical model

To test hypothesis 1, examining the impact of agricultural insurance on food safety, drawing on the by Liu Wei et al. (14), we constructed the following static panel model.

(1) $$\begin{array}{l} \mathrm{Diseve}:food_{\mathrm{it}}=\alpha_{0}+\alpha_{1}\mathrm{Insurance}:per_{\mathrm{it}}+\\ \;\alpha_{2}\mathrm{Control}s_{\mathrm{it}}+\mu_{i}+r_{t}+\varepsilon_{\mathrm{it}} \end{array}$$

Here, *i* represents the province, *t* represents the year, *Diseve*_*food_it_* represents the number of foodborne disease events, *Insurance*_*per_it_* represents *per capita* agricultural insurance premium income; *Controls_it_* represents control variables, *μ_i_* represents province fixed effects, *r_t_* represents time fixed effects, and $\varepsilon_{\mathrm{it}}$ represents the random error term. We mainly focus on the impact coefficient *α*_1_ of *per capita* agricultural insurance premium income on the number of foodborne disease events. If the sign of *α*_1_ is negative, it indicates that agricultural insurance can reduce the incidence rate of foodborne diseases and enhance food safety, thereby testing Hypothesis 1.

To test hypothesis 2, examining whether agricultural insurance enhances food safety through improving agricultural technological innovation and reducing environmental pollution. drawing on the research by Wen Zhonglin (53), Liu Yiwen et al. (54), we constructed the following mediation effect model.

(2) $$\begin{array}{l} M_{\mathrm{it}}=\beta_{0}+\beta_{1}\mathrm{Insurance}:per_{\mathrm{it}}+\\ \;\beta_{2}\mathrm{Control}s_{\mathrm{it}}+\mu_{i}+r_{t}+\varepsilon_{\mathrm{it}} \end{array}$$

(3) $$\begin{array}{l} \mathrm{Diseve}:food_{\mathrm{it}}=\lambda_{0}+\lambda_{1}\mathrm{Insurance}:per_{\mathrm{it}}+\\ \;\lambda_{2}M_{\mathrm{it}}+\lambda_{3}\mathrm{Control}s_{\mathrm{it}}+\\ \;\mu_{i}+r_{t}+\varepsilon_{\mathrm{it}} \end{array}$$

Here, *M_it_* = {*Innovationit*, *Pollutionit*}, *Innovation_it_* represents agricultural technological innovation, *Pollution_it_* represents the environmental pollution index, and the meanings of other variables are as above. We focus on the impact coefficient *β*_1_ of *per capita* agricultural insurance premium income on the mediator variables, the impact coefficient *λ*_1_ of *per capita* agricultural insurance premium income on the number of foodborne disease events after including mediator variables, and the impact coefficient *λ*_2_ of mediator variables on the number of foodborne disease events. If the estimated coefficients *β*_1_ and *λ*_2_ are significant, it means the mediation effect exists. Observing *λ*_1_, if *λ*_1_ is significant, then the mediator variables play a partial mediating role; if *λ*_1_ is not significant, then the mediator variables play a complete mediating role. Thus, when the estimated coefficients *β*_1_, *λ*_1_ and *λ*_2_ are significant, it indicates that the impact of agricultural insurance on food safety exists through two mechanisms: agricultural technological innovation and environmental pollution, and Hypothesis 2 is supported.

To test hypothesis 3, examining whether consumer confidence has a moderating effect on the impact of agricultural insurance, we constructed the following moderating effect model.

$$\begin{array}{l} \mathrm{Diseve}:food_{\mathrm{it}}=\theta_{0}+\theta_{1}\mathrm{Insurance}:per_{\mathrm{it}}\times \\ \;\mathrm{Consume}r_{t}+\theta_{2}\mathrm{Insurance}:per_{\mathrm{it}} \end{array}$$

(4) $$+\theta_{3}\mathrm{Consume}r_{t}+\theta_{4}\mathrm{Control}s_{\mathrm{it}}+\mu_{i}+r_{t}+\varepsilon_{\mathrm{it}}$$

Here, *Consumer_t_* represents the consumer confidence index, and the meanings of other variables are as above. We focus on the coefficient *θ*_1_ of the interaction term between *per capita* agricultural insurance premium income and consumer confidence index. If the estimated coefficient *θ*_1_ is significant, it means the moderating effect exists. If *θ*_1_ has the same sign as *α*_1_ in equation (1), it suggests that the enhancement of consumer confidence has a positive promotional effect on the impact of agricultural insurance. Hypothesis 3 is supported.

### 3.2 Variables selection

#### 3.2.1 Dependent variable

Drawing on the research of Li (55) and Fulisha and Qin (56) *per capita* agricultural insurance premium income ( Insurance:per ) is used as a proxy variable for agricultural insurance. Meanwhile, the depth of agricultural insurance ( Insurance:dep ) is used as an alternative variable for robustness tests. *Per capita* agricultural insurance premium income is represented by the ratio of agricultural insurance premium income to the rural population, and the depth of agricultural insurance is represented by the ratio of agricultural insurance premium income to the added value of the primary industry.

#### 3.2.2 Independent variables

Following the study of Chen et al. (57) the number of foodborne disease events ( Diseve:food ) is used as a proxy variable for food safety. Simultaneously, the number of foodborne disease patients ( Dispat:food ) is used as an alternative variable for robustness tests.

#### 3.2.3 Mediating variables

Following the study of Wang et al. (58) and Kong et al. (59) this paper uses agricultural technological innovation ( Innovation ) and environmental pollution index ( Pollution ) as mediating variables. Agricultural technological innovation is represented by the number of patents for agricultural technological innovation, and the environmental pollution index is represented by a composite index synthesized from three indicators: industrial sulfur dioxide emissions, industrial smoke (dust) emissions, and industrial wastewater discharge, using the entropy weight method.

#### 3.2.4 Moderating variable

Following the study of Wang et al. (60) the Chinese Consumer Confidence Index ( Consumer ) is used as a moderating variable in this paper.

#### 3.2.5 Control variables

Drawing on the research by Jiao et al. (61), the control variables used in this study are as follows: (1) Fiscal support for agriculture expenditure ( Fin ), represented by the ratio of government fiscal expenditure on agriculture, forestry, and water affairs to total fiscal expenditure; (2) Urbanization level ( Urb ), represented by the proportion of urban population; (3) Level of agricultural mechanization ( Mech ), represented by the total power of agricultural machinery; (4) Regional economic level ( Gdp ), represented by the GDP growth rate.

### 3.3 Data sources

This paper selects annual data from 34 provincial-level administrative regions in China from 2011 to 2021 as the initial sample and processes it as follows: (1) Excluding the four regions with severe missing data: Hong Kong Special Administrative Region, Macao Special Administrative Region, Taiwan Province, and Tibet Autonomous Region. (2) To eliminate the influence of extreme values, Winsorize tailoring is applied to the main continuous variables at the 1 and 99% quantiles. The final research sample includes 30 provincial-level administrative regions. The agricultural insurance data are sourced from the “China Insurance Yearbook,” foodborne disease data from the “China Health and Wellness Statistical Yearbook,” agricultural-related data from the “China Rural Statistical Yearbook,” agricultural technological innovation data from the CNKI patent database, and other data from the “China Statistical Yearbook.” Table 1 reports the descriptive statistical results of the main variables. Within the sample period, the standard deviation of the number of foodborne disease events is 207.3, with a minimum value of 2 and a maximum value of 1,221. The standard deviation of *per capita* agricultural insurance premium income is 115.7, with a minimum value of 4.903 and a maximum value of 562.0. It can be seen that the fluctuation range of foodborne diseases and *per capita* insurance premium income is relatively large, indicating the large difference between different provinces.

**Table 1:** Descriptive statistics of the variables.

Variable	N	Mean	Std. Dev.	Min	Max
Diseve_food	330	122.3	207.3	2	1,221
Insurance_per	330	116.5	115.7	4.903	562.0
Fin	330	11.41	3.295	4.318	18.63
Urb	330	59.60	12.11	37.25	89.30
Mech	330	3,420	2,906	102.7	12,400
Gdp	330	0.0931	0.0671	−0.200	0.244
Innovation	330	2,879	2,982	78	13,400
Pollution	330	0.232	0.143	0.0168	0.650
Consumer	330	112.6	9.672	100.5	126.6

## 4 Empirical results

### 4.1 Baseline results

Table 2 reports the regression results of the impact of agricultural insurance ( Insurance:per ) on foodborne diseases ( Diseve:food ). To demonstrate the robustness of the estimation results, control variables are gradually included in the econometric model (1), and each regression equation controls for province fixed effects and time fixed effects. In column (1), the coefficient estimate is −0.4611 and significant at the 1% level, indicating that higher *per capita* agricultural insurance premium income is associated with a lower incidence rate of foodborne diseases, significantly enhancing food safety. Furthermore, after sequentially adding control variables in columns (2) and (3), the sign and significance of the estimated coefficient remain unchanged, thereby supporting the validity of Hypothesis 1.

**Table 2:** Benchmark Regression results.

Variable	(1)	(2)	(3)
	Diseve_food	Diseve_food	Diseve_food
Insurance_per	−0.4611*** (0.1571)	−0.3852*** (0.1358)	−0.3430*** (0.1176)
Control variable	No	Part	Yes
Time fixed effect	Yes	Yes	Yes
Individual fixed effects	Yes	Yes	Yes
Observations	330	330	330
R-squared	0.3521	0.3854	0.4018

1. Significance levels: ***p < 0.001; **p < 0.01; *p < 0.1. 2. To improve the comparability and interpretability of the data, All the variables were processed for normalization.

### 4.2 Mediation effect

Table 3 reports the mediation effect of agricultural technological innovation ( Innovation ) and environmental pollution ( Pollution ) on the mechanism through which agricultural insurance enhances food safety. In column (1), the estimated coefficient value for *per capita* agricultural insurance premium income ( Insurance:per ) is 0.4344, significant at the 5% level, indicating that agricultural insurance can promote agricultural technological innovation. Simultaneously, in column (2), the estimated coefficient for agricultural technological innovation ( Innovation ) is significantly negative, and the estimated coefficient for *per capita* agricultural insurance premium income is significantly negative, suggesting that agricultural insurance can reduce the incidence rate of foodborne diseases and enhance food safety by improving agricultural technological innovation, verifying the partial mediation effect of agricultural technological innovation. In column (3), the estimated coefficient value for *per capita* agricultural insurance premium income ( Insurance:per ) is −0.2079, significant at the 1% level, indicating that agricultural insurance can reduce the level of environmental pollution. In column (4), the estimated coefficient for environmental pollution ( Pollution ) is significantly positive, and the estimated coefficient for *per capita* agricultural insurance premium income ( Insurance:per ) is significantly negative, meaning that agricultural insurance can reduce environmental pollution, thereby decreasing the incidence rate of foodborne diseases and enhancing food safety, verifying the partial mediation effect of environmental pollution. Overall, agricultural insurance can enhance food safety by improving agricultural technological innovation and reducing the level of environmental pollution, thereby supporting Hypothesis 2.

**Table 3:** Results of regression of mediation and moderating effect.

Variable	(1)	(2)	(3)	(4)	(5)
	Innovation	Diseve_food	Pollution	Diseve_food	Diseve_food
Insurance_per	0.4344** (0.1796)	−0.3865** (0.1622)	−0.2079*** (−2.9081)	−0.3812*** (0.1166)	−0.1514 (0.1084)
Innovation		−0.2202* (0.1141)			
Pollution				0.2110** (0.1030)	
Consumer × Insurance_per					−0.1450** (0.0694)
Control variable	Yes	Yes	Yes	Yes	Yes
Time fixed effect	Yes	Yes	Yes	Yes	Yes
Individual fixed effects	Yes	Yes	Yes	Yes	Yes
Observations	330	330	330	330	330
*R*-squared	0.4767	0.3720	0.1772	0.3979	0.4022

1. Significance levels: ***p < 0.001; **p < 0.01; *p < 0.1. 2. All the variables were processed for normalization.

### 4.3 Moderating effect

Table 3 reports the regression results of introducing the interaction term of consumer confidence index ( Consumer ) and *per capita* insurance premium income ( Insurance:per ) into the baseline model, examining the moderating effect of consumer confidence on the impact of agricultural insurance. In column (4), the estimated coefficient for the interaction term between *per capita* insurance premium income ( Insurance:per ) and consumer confidence ( Consumer ) is −0.1450, significant at the 5% level. Simultaneously, in column (3) of Table 1, the estimated coefficient for *per capita* insurance premium income ( Insurance:per ) is significantly negative at the 1% level, with the same sign as the interaction term coefficient, meaning that an increase in consumer confidence has a positive moderating effect on the impact of agricultural insurance. That is, the higher the consumer confidence, the more significant the effect of agricultural insurance in enhancing food safety.

### 4.4 Heterogeneity analysis

Table 4 reports the regression results of the heterogeneity analysis. In provinces with more developed agriculture, the positive impact of agricultural insurance on food safety may be more significant. This hypothesis is based on the fact that the agricultural production systems in these areas are usually more mature and complex, with a higher dependence on stable production conditions and risk management mechanisms. In these provinces, the scale and specialization of agricultural production are higher, and the types and degrees of risks that production may face are relatively more complex, thus farmers have a higher demand and reliance on agricultural insurance as a risk management tool (62). By providing financial compensation, agricultural insurance mitigates losses caused by unpredictable factors such as natural disasters and market fluctuations, allowing agricultural producers to maintain the stability of production activities, thereby helping to ensure the continuity of the food supply chain and food safety.

**Table 4:** Analysis of heterogeneity.

Variable	Level of agricultural development		Fiscal support for agriculture	
	Below median	Above median	Below median	Above median
	(1)	(2)	(3)	(4)
Insurance_per	−0.2490** (0.0974)	−0.5390** (0.2117)	−0.1818** (0.0726)	−0.4851** (0.2104)
Control variable	Yes	Yes	Yes	Yes
Time fixed effect	Yes	Yes	Yes	Yes
Individual fixed effects	Yes	Yes	Yes	Yes
Observations	165	165	165	165
*R*-squared	0.4424	0.5036	0.5827	0.4471

1. Significance levels: ***p < 0.001; **p < 0.01; *p < 0.1. 2.All the variables were processed for normalization.

To verify this theoretical inference, we divided the whole sample into groups with higher and lower levels of agricultural development, specifically: using the ratio of agricultural output value to regional total output value to measure the level of agricultural development, provinces above the median were classified as having a higher level of agricultural development, and those below the median were classified as having a lower level. Columns (1) and (2) of Table 4 show that the absolute value of the estimated coefficient for the group with a higher level of agricultural development is greater than that for the group with a lower level of agricultural development (0.5390 > 0.2490), indicating that the impact of agricultural insurance on improving food safety is more pronounced in provinces with a higher level of agricultural development.

In exploring the impact of agricultural insurance on food safety, this study posits a theoretical hypothesis that in provinces with greater policy support, the positive effect of agricultural insurance on food safety may be more evident. This hypothesis is based on the recognition that government policy support can significantly promote the popularity and efficiency of agricultural insurance products, enhancing their application in agricultural production by reducing insurance costs, improving compensation efficiency, and promoting agricultural insurance knowledge, thereby more effectively ensuring the stability and safety of the food production process (63). Policy support includes not only direct financial subsidies but also policy formulation and implementation, such as developing regulations and policies favorable to the development of agricultural insurance, providing agricultural insurance training, etc., all of which help to increase the coverage and compensation efficiency of agricultural insurance, thereby positively affecting food safety.

To verify this theoretical inference, we divided the whole sample into groups with greater and lesser fiscal support for agriculture, specifically: using the ratio of government fiscal expenditure on agriculture, forestry, and water affairs to total fiscal expenditure to measure the intensity of fiscal support for agriculture, provinces above the median were classified as having greater fiscal support for agriculture, and those below the median were classified as having lesser fiscal support. Columns (3) and (4) of Table 4 show that the absolute value of the estimated coefficient for the group with greater fiscal support for agriculture is greater than that for the group with lesser fiscal support (0.4851 > 0.1818), indicating that the impact of agricultural insurance on improving food safety is more pronounced in provinces with greater fiscal support for agriculture.

### 4.5 Robustness tests

To verify the reliability of the baseline regression results, this paper conducted a series of robustness tests from multiple dimensions.

#### 4.5.1 Addressing endogeneity issues

Generally, bidirectional causality, omission of important explanatory variables, and measurement bias in core explanatory variables can lead to endogeneity issues in the model. Firstly, regarding the bidirectional causal relationship, agricultural insurance is a variable of national economic policy, and it is unlikely that diseases at the individual level could influence national policy. Thus, the existence of a bidirectional causal relationship between agricultural insurance and food safety can essentially be ruled out. Moreover, for the latter two scenarios, the systematic GMM method for identifying instrumental variables within the model and the instrumental variable method can resolve issues related to omitted variables and measurement bias to the greatest extent possible. Therefore, this paper conducts robustness tests using two methods: establishing a dynamic panel data econometric model and the instrumental variable method.

##### 4.5.1.1 Further test based on dynamic panel data econometric model

We introduce the first lag of the dependent variable into econometric model (1) to establish the following dynamic panel model:

(5) $$\begin{array}{l} \mathrm{Diseve}:food_{\mathrm{it}}=\alpha_{0}+\mathrm{\rho LDiseve}:food_{\mathrm{it}-1}+\\ \;\alpha_{1}\mathrm{Insuranc}{e_{\mathrm{per}}}_{\mathrm{it}}+\alpha_{2}\mathrm{Control}s_{\mathrm{it}}+\\ \;\mu_{i}+r_{t}+\varepsilon_{\mathrm{it}} \end{array}$$

$\mathrm{LDiseve}:food_{\mathrm{it}-1}$ represents the first lag of the number of foodborne disease events, *ρ* is the estimated coefficient of the lag term, and the meanings of other variables are as above. The dynamic panel data econometric model mainly uses two estimation methods: Difference GMM (DIF-GMM) and System GMM (SYS-GMM). To ensure the reliability of the research conclusions, both methods are used for regression.

Columns (1) and (2) of Table 5 are the regression results of the dynamic panel model, where column (1) is the regression result of the Difference GMM method, and column (2) is the regression result of the System GMM method. It can be seen that the *p*-values of the AR(1) test are all less than 0.1, rejecting the null hypothesis, indicating that there is first-order autocorrelation in the residual terms. The p-values of the AR(2) test are all greater than 0.1, accepting the null hypothesis, indicating that there is no second-order autocorrelation in the residual terms. The Sargan test values are all greater than 0.1, unable to reject the null hypothesis that the instrumental variables are valid, indicating that the selection of instrumental variables is reasonable. The above test results suggest that there is no issue of second-order serial correlation and over-identification in the model setup, verifying the rationality of the model setup. Looking at the regression coefficients of variables, the estimated coefficient of *per capita* agricultural insurance premium income remains negative at the 1% level, meaning that the baseline regression results do not depend on a specific econometric model method, indicating that the conclusions of this paper are robust.

**Table 5:** Endurance test.

Variable	DIF-GMM	SYS-GMM	IV-2SLS
	(1)	(2)	(3)
	Diseve_food	Diseve_food	Diseve_food
Insurance_per	−0.1389*** (0.0294)	−0.1192*** (0.0222)	−0.3292** (0.1558)
L.Diseve_food	0.4726*** (0.0137)	0.7992*** (0.0240)	
Control variable	Yes	Yes	Yes
Observations	270	300	329
*R*-squared			0.3248
Observations	270	300	329
AR (1)	0.0386	0.0263	
AR (2)	0.3886	0.4752	
Sargan	1.0000	1.0000	
The *p*-value of the Anderson Canon Correlation LM statistic			0.0000
Cragg–Donald Wald *F* statistic			49.132 [16.38]

1. Significance levels: ***p < 0.001; **p < 0.01; *p < 0.1. 2. All the variables were processed for normalization.

##### 4.5.1.2 Addressing endogeneity issues using the instrumental variable method

Considering that agricultural insurance is a type of property insurance (64, 65), which is highly related to the level of development of property insurance and that the level of property insurance development cannot directly affect food safety. Therefore, we selected *per capita* property insurance premium income as an instrumental variable and conducted robustness tests using the panel instrumental variable 2SLS method.

Column (3) of Table 5 presents the regression results of the instrumental variable 2SLS method. It can be seen that, in the relevancy test of the instrumental variable, the *p*-value of the Anderson canonical correlation LM statistic is less than 0.1, rejecting the null hypothesis of insufficient instrumental variable identification. The Cragg-Donald Wald F statistic is greater than the corresponding Stock-Yogo critical value of 16.38, rejecting the null hypothesis of a weak instrumental variable, indicating that the selection of the instrumental variable is appropriate. The estimated coefficient of *per capita* agricultural insurance premium income remains negative at the 1% level, consistent with the baseline results, meaning that the research conclusions obtained in this paper are reliable.

#### 4.5.2 Variable substitution

This study conducted robustness tests by substituting the core explanatory variables and changing the measurement methods of the dependent variables to further verify the reliability of the baseline regression results. First, we used the variable of agricultural insurance depth in place of *per capita* agricultural insurance premium income for regression analysis. The results are shown in column (1) of Table 6, where the estimated coefficient value for agricultural insurance depth is −0.4594, significant at the 1% level. Secondly, we substituted the number of foodborne disease patients for the number of foodborne disease events for regression analysis. The results are shown in column (2) of Table 6, where the estimated coefficient for *per capita* agricultural insurance premium income remains significantly negative. The consistency and significance of these regression results with the baseline regression demonstrate that the conclusions of this paper are robust.

**Table 6:** Robustness test.

Variable	(1)	(2)	(3)
	Diseve_food	Dispat_food	Diseve_food
Insurance_per		−0.2826*** (0.0703)	−0.2561*** (0.0878)
Insurance_dep	−0.4594*** (0.1598)		
Control variable	Yes	Yes	Yes
Time fixed effect	Yes	Yes	Yes
Individual fixed effects	Yes	Yes	Yes
Observations	330	330	210
*R*-squared	0.3943	0.4151	0.2909

1. Significance levels: ***p < 0.001; **p < 0.01; *p < 0.1. 2. All the variables were processed for normalization.

#### 4.5.3 Subsample interval estimation

Currently, as the development of agricultural insurance gradually matures and gains attention, and since 2015, the Ministry of Agriculture and Rural Affairs has increasingly focused on the use of pesticides and fertilizers, the “double reduction” policy for pesticides and fertilizers has also received significant attention. Considering the potential impact of the “double reduction” policy on pesticides and fertilizers on the baseline results of this paper, to ensure the principle of random sample selection, this paper uses the subsample interval method, excluding sample data from 2007 to 2014 for robustness tests. As can be seen from column (3) of Table 6, the estimated coefficient for *per capita* agricultural insurance premium income remains significant at the 1% level, and the sign direction is consistent with the baseline regression, indicating that the baseline results of this paper have good robustness.

## 5 Conclusion

Through an in-depth analysis of annual data from 30 provinces in China from 2011 to 2021, we tested the impact of agricultural insurance on the safety of food consumed by consumers. The research results show that agricultural insurance can significantly reduce the incidence rate of foodborne diseases and enhance food safety. Further mechanism tests reveal that agricultural insurance can effectively improve food safety through two main channels—enhancing agricultural technological innovation and reducing environmental pollution. At the same time, tests of the moderating effect show that enhanced consumer confidence has a positive promotional effect on the impact of agricultural insurance. Notably, the impact of agricultural insurance on enhancing food safety is more pronounced in provinces with a higher level of agricultural development and greater governmental support for agriculture.

Although this study provides new insights into the impact of agricultural insurance on food safety, we also recognize some limitations of the research. Firstly, despite our efforts to control for the multiple possible interfering factors, there may still be unobserved variables affecting the relationship between agricultural insurance and food safety. Besides, this study primarily focuses on provincial panel data, which may not fully reveal the subtle differences between prefecture-level cities within the regions.

Combining theoretical analysis and empirical results, this paper proposes the following policy recommendations to further optimize the impact of agricultural insurance policies on food safety.

Strengthen the integration of agricultural technological innovation and insurance. The government should promote agricultural technological innovation through policy incentives and financial support, especially those technologies that can directly improve food safety. Simultaneously, encourage insurance companies to develop insurance products specifically for agricultural production methods that adopt new technologies, reducing the risk for farmers to adopt new technologies and accelerating the application of technology in agricultural production.

Promote environmentally friendly agricultural production methods. The government should formulate and implement a series of policy measures to encourage farmers to adopt environmentally friendly agricultural production methods, such as organic farming, water-saving irrigation technologies, etc. Insurance companies should provide corresponding insurance products, offering more attractive insurance terms for farmers who adopt these production methods, thereby reducing the negative impact of agricultural production on the environment and enhancing food safety.

Increase consumer awareness of the role of agricultural insurance. Through media, public education, and other channels, intensify the promotion of the role of agricultural insurance in enhancing food safety, raising consumer awareness of the importance of agricultural insurance. This can not only enhance consumer confidence but also encourage more agricultural producers to participate in agricultural insurance programs, forming a virtuous cycle.

By implementing the above policy recommendations, agricultural insurance policies can be further optimized to play a greater role in enhancing food safety. This will not only provide safer food for consumers but also promote the sustainable development of agricultural production, offering strong policy support for achieving the dual goals of food safety and sustainable agricultural development.

## Data availability statement

The original contributions presented in the study are included in the article/supplementary material, further inquiries can be directed to the corresponding author.

## Author contributions

PR: Conceptualization, Data curation, Formal analysis, Funding acquisition, Investigation, Methodology, Project administration, Resources, Software, Supervision, Validation, Visualization, Writing – original draft, Writing – review & editing. SY: Data curation, Project administration, Resources, Writing – review & editing. YZ: Resources, Visualization, Writing – review & editing.
